# Rhabdomyolysis and Resultant Acute Renal Failure due to Legionella Pneumonia in a Patient with Human Immunodeficiency Virus

**DOI:** 10.1155/2023/8772577

**Published:** 2023-12-23

**Authors:** Margaret Kypreos, Roma Mehta

**Affiliations:** Division of Pulmonary and Critical Care Medicine, University of Texas Southwestern Medical Center, Dallas, TX, USA

## Abstract

Legionnaires' disease is a severe pneumonia caused by *Legionella* that results in laboratory abnormalities including hyponatremia and elevated liver enzymes. Rarely skeletal muscle and renal abnormalities occur. This case report describes a case of *Legionella* pneumonia complicated by rhabdomyolysis and acute renal failure in a patient with the human immunodeficiency virus.

## 1. Introduction

Rhabdomyolysis is a syndrome due to the injury of skeletal muscle fibers that leads to release of myoglobin, creatine kinase, and lactate dehydrogenase into circulation [1]. Etiologies include trauma, medications, infections, toxins, extreme physical exertion, temperature extremes, and hereditary as well as acquired metabolic myopathies [1, 2]. Complications include acute kidney injury, disseminated intravascular coagulopathy, and acute compartment syndrome. The pathophysiology of the acute kidney injury is thought to be secondary to myoglobin via three different mechanisms. These include renal vasoconstriction, formation of intratubular casts, and direct toxicity of myoglobin to kidney tubular cells [3]. Bacterial as well as viral infections can lead to the development of rhabdomyolysis, and Betrosian et al. observed that Gram-positive infections were the more frequent etiology [4]. On the contrary, Kumar et al. observed that Gram-negative infections were the leading cause of rhabdomyolysis; however, this was thought to be partially explained by the high proportion of diabetic patients in that particular cohort. Viral infectious etiologies include influenza, human immunodeficiency virus (HIV), Coxsackie virus, Epstein–Barr virus, cytomegalovirus, herpes simplex virus, varicella zoster virus, and West Nile virus [2]. Here, we report a case of rhabdomyolysis and acute renal failure due to *Legionella* pneumonia in a HIV patient.

## 2. Case Report

A 60-year-old male with a past medical history of HIV diagnosed thirty-four years ago with a CD4 count of 344, bipolar disorder, and asthma presented with a six-day history of fever, cough, and shortness of breath as well as a four-day history of nausea, vomiting, and diarrhea that started after consumption of shellfish. He was found to be in acute hypoxemic respiratory failure requiring 3 L of supplemental oxygen. Complete metabolic panel revealed hyponatremia with a sodium level of 132 mmol/L, stage 1 acute kidney injury with a creatinine level of 1.63 mg/dL, and an elevated aspartate transaminase level of 224 U/L. Computed tomography of the chest showed a consolidation in the left upper lobe and *Lingula*, thus initiating ceftriaxone and doxycycline. Stool culture, blood cultures, clostridium difficile polymerase chain reaction, influenza, respiratory syncytial virus, and novel coronavirus were negative. Urine *Legionella* was then obtained and found to be positive on hospital day 2, and thus antibiotic therapy was then changed to levofloxacin monotherapy. On hospital day 3, oxygen requirements rapidly increased to 10 L nasal cannula, and thus he was transferred to the intensive care unit. Urine output began to decrease, and a complete metabolic panel showed a rising creatinine of 5.03 mg/dL, aspartate transaminase of 3,118 U/L, and alanine transaminase of 386 U/L. Antibiotics were broadened to include vancomycin, cefepime, and levofloxacin. Nephrology was consulted, and urinalysis was notable for microscopic hematuria, proteinuria, and sterile pyuria. Urine sediment was notable for muddy brown casts consistent with acute tubular necrosis. Creatine kinase level was obtained given the rising aspartate transaminase that was out of proportion to the alanine transaminase and found to be elevated at 224,100 U/L. Etiology of the rhabdomyolysis was thought to be secondary to the *Legionella* infection. Continuous intravenous fluids were initiated, however discontinued when volume overload occurred and oliguria persisted leading to the initiation of hemodialysis on hospital day 6. Vancomycin was discontinued, and cefepime was changed to piperacillin-tazobactam on hospital day 7 given altered mental status. Oxygen requirements decreased, and he was transferred out of the intensive care unit on hospital day 9. Piperacillin-tazobactam course was completed on hospital day 10, and a tunneled dialysis catheter was placed on hospital day 14. Creatine kinase level decreased after initiation of hemodialysis and was found to be 1,688 by hospital day 13. The course of levofloxacin was completed on hospital day 17, and he was discharged home after a total of 23 days in the hospital.

## 3. Discussion

*Legionella* is the second most common cause of pneumonia that leads to admission in the intensive care unit [5]. These bacteria are typically found in bodies of freshwater but can also be present in human-made water systems such as cooling towers [6]. Transmission occurs via inhalation of contaminated water droplets from a particular source which, interestingly, was unable to be identified in this case. It is possible that the consumed shellfish was washed with contaminated water; however, no outbreak was noted after this case. Legionnaire's disease can present with primary pulmonary manifestations as well as rare extra pulmonary manifestations of rhabdomyolysis and acute renal failure. The overall mortality is approximately 54%; however, a 38% mortality is associated if the triad of pneumonia, rhabdomyolysis, and acute renal failure is present [5]. Rhabdomyolysis secondary to *Legionella* was first described by Posner et al. in 1980, and since then, just over twenty case reports have been published that describe the association of rhabdomyolysis and acute renal failure [5]. The mechanism of action of rhabdomyolysis in this infection is thought to be secondary to the release of an endotoxin given that biopsy specimens are negative for the organism. It is hypothesized that the endotoxin leads to local ischemia-induced changes from vasoconstrictive effects on the small blood vessels [5]. Alternate theories include direct invasion of the bacteria into the muscle; however, that is usually seen with the influenza virus [6]. Rhabdomyolysis can lead to acute kidney injury by the occlusion of distal tubules with myoglobin casts; however, direct renal injury from the bacteria itself is also possible [5]. Shah et al. demonstrated the presence of *Legionella* pneumophila by immunofluorescence microscopy on a kidney biopsy, thus supporting the latter mechanism of action [7]. Tubulointerstitial nephritis has also been reported as an etiology of renal dysfunction [5, 8]. In this case, the patient had a history of HIV and was compliant with a home regimen of darunavir and emtricitabine/tenofovir, as well as ritonavir. Rhabdomyolysis commonly occurs in the HIV population with an increased risk in those with advanced disease. A cohort study from Kaiser Permanente showed that the risk of rhabdomyolysis is higher in HIV-positive patients, especially those with a CD4 count less than 200, an HIV RNA load greater than 500 copies per ml, and substance abuse. Antiretroviral use and certain antiretroviral classes were not associated with an increased risk of rhabdomyolysis in this study [9]. Etiologies other than HIV infection itself are also associated with rhabdomyolysis and include infection, adverse medication effects, drug interactions, alcohol, and illicit drug use. Infection is the most common etiology with respiratory infections accounting for 38% of all cases. Four out of the seven patients in the Joshi and Liu case series had documented infections with Mycoplasma pneumonia as the respiratory case [10]. The majority of these patients were not on antiretroviral therapy; however, substance abuse was common. Alcohol abuse was identified in five out of the seven patients, and illicit drug use was identified in three of the patients [10]. Coadministration with other medications that can cause rhabdomyolysis should be considered as a potentiating factor for the development especially if there is interaction with cytochrome P450 (CYP) or P glycoprotein [11]. Antiretroviral medications can be substrates, inhibitors, and inducers of CYP. Koubar et al. identified statins as the most common medication leading to rhabdomyolysis in HIV patients, and the increased prevalence was thought to be secondary to dyslipidemia and atherosclerotic disease associated with antiretroviral use. Drug interactions between statins and the CYP3A4 inhibitors ritonavir and cobicistat were also noted [12]. In our particular case, the patient was taking an antiretroviral regimen that included tenofovir; however, this was not newly initiated. In addition, a statin was not part of his medication regimen, and his CD4 count was greater than 200. Antiretroviral therapy alone rarely leads to rhabdomyolysis. Spiegel et al. reported a case of severe rhabdomyolysis in an HIV-positive patient after initiation of tenofovir and subsequently abacavir [13]. The development of rhabdomyolysis occurred just days after the initiation of these regimens, and acute tubular necrosis secondary to rhabdomyolysis was confirmed on kidney biopsy after initiation of tenofovir. A subsequent biopsy was not performed after the recurrent event with initiation of abacavir; however, this was suspected to be the etiology as no other risk factors were present. Tenofovir does result in nephrotoxicity via various mechanisms including tubular injury leading to acute tubular necrosis, Fanconi syndrome, diabetes insipidus, and nephrolithiasis. However, rhabdomyolysis is not a common presentation thus making the above case a unique manifestation [13]. Quinolone-induced rhabdomyolysis is rare, however has been reported for approximately two decades. The mechanism of action is unclear, but a theory of vascular hyperpermeability has been suggested [14]. Prior case reports have shown the adverse event to occur within six days of antibiotic administration with an increased occurrence in patients with baseline renal impairment [11, 14]. The predominant enzyme involved in antiretroviral medication is CYP3A4 which is not affected by levofloxacin thus decreasing the possibility of potentiation with concomitant use [15]. In our case, the elevated creatine kinase was found to be elevated one day after initiation of levofloxacin; however, aspartate aminotransferase was elevated in isolation since the day of admission suggesting that rhabdomyolysis was already occurring. Given this, the *Legionella* infection was likely the leading etiology of his rhabdomyolysis; however, other risk factors were present that could have increased the subsequent development.

## 4. Conclusion

*Legionella* pneumonia can be complicated by rhabdomyolysis as well as acute renal failure. Even though these manifestations are rare, this infectious etiology should be considered in a patient presenting with acute hypoxemic respiratory failure, acute renal failure, and an elevated creatine kinase level.

## Data Availability

All of the data are available as part of the article and there is no additional source data.
